# 格菲妥单抗联合维泊妥珠单抗治疗9例CAR-T细胞治疗失败的复发/难治弥漫大B细胞淋巴瘤疗效及安全性分析

**DOI:** 10.3760/cma.j.cn121090-20251219-00604

**Published:** 2026-07

**Authors:** 欢 张, 小圆 贺, 霞 肖, 海容 吕, 明峰 赵

**Affiliations:** 天津市第一中心医院血液科，天津市血栓与止血研究所，天津 300192 Department of Hematology, Tianjin First Central Hospital, Tianjin Thrombosis and Hemostasis Institute, Tianjin 300192, China

## Abstract

为探讨格菲妥单抗联合维泊妥珠单抗治疗嵌合抗原受体T（CAR-T）细胞治疗失败的复发/难治弥漫大B细胞淋巴瘤（R/R DLBCL）疗效及安全性，纳入2024年3月至2025年4月期间在天津市第一中心医院血液科接受格菲妥单抗联合维泊妥珠单抗治疗的CAR-T细胞治疗失败的9例R/R DLBCL患者，对其临床资料进行回顾性分析。6个周期后患者总缓解率为77.8％，完全缓解率为55.6％。中位随访12（5～18）个月，1年无进展生存率和总生存率分别为71.1％（95％ *CI*：56.3％～86.1％）和76.2％（95％ *CI*：64.0％～94.2％）。9例患者均发生细胞因子释放综合征，其中7例为1～2级、2例为3级。1例发生1级免疫效应细胞相关神经毒性综合征。格菲妥单抗联合维泊妥珠单抗治疗CAR-T细胞治疗失败的R/R DLBCL显示出初步疗效且安全性可控。

## Abstract (en)

To evaluate the efficacy and safety of glofitamab combined with polatuzumab vedotin in the treatment of relapsed/refractory diffuse large B-cell lymphoma（R/R DLBCL）after failure of chimeric antigen receptor T（CAR-T）cell therapy. Nine patients with R/R DLBCL who failed CAR-T therapy and received glofitamab combined with polatuzumab vedotin treatment at the Hematology Department of Tianjin First Central Hospital from March 2024 to April 2025 were selected for retrospective analysis of their clinical data. The overall response rate was 77.8％, and the complete response rate was 55.6％ following six cycles of treatment. The median follow-up period was 12（5–18）months. The 1-year progression-free survival rate and overall survival rate were 71.1％（95％ *CI*: 56.3％–86.1％）and 76.2％（95％ *CI*: 64.0％–94.2％）, respectively. All 9 patients experienced cytokine release syndrome, with 7 cases at grade 1–2 and 2 cases at grade 3. One patient had grade 1 immune effector cell-associated neurotoxicity syndrome. Glofitamab combined with polatuzumab vedotin shows preliminary efficacy and a manageable safety in patients with R/R DLBCL after CAR-T cell therapy failure.

嵌合抗原受体T（CAR-T）细胞治疗在复发/难治弥漫大B细胞淋巴瘤（R/R DLBCL）中取得了令人鼓舞的疗效[1]–[2]。CAR-T细胞治疗R/R DLBCL的总体缓解率（ORR）为50％～80％，完全缓解（CR）率为40％～60％[3]–[5]。然而长期随访中患者CAR-T细胞治疗后复发/进展成为新的挑战，CAR-T细胞治疗失败的R/R DLBCL患者生存率极低，缺乏有效治疗手段[6]–[7]。格菲妥单抗是一种靶向CD3×CD20的全人源化IgG双特异性抗体，该抗体能够识别T细胞上的CD3，且能与B细胞上的肿瘤抗原CD20结合，介导免疫效应，最终诱导肿瘤细胞裂解死亡[8]。维泊妥珠单抗是一种由高细胞毒性的单甲基澳瑞他汀E（MMAE）偶联到抗CD79b抗体的药物偶联物[9]。既往格菲妥单抗单药治疗方案在CAR-T细胞治疗失败的R/R DLBCL中取得一定疗效[10]。格菲妥单抗与维泊妥珠单抗的联合具有协同增效的机制[11]。本研究分析CAR-T细胞治疗失败的R/R DLBCL患者应用格菲妥单抗联合维泊妥珠单抗治疗的安全性和有效性。

## 病例与方法

1. 病例：本研究为单中心回顾性研究，纳入2024年3月至2025年4月期间在天津市第一中心医院血液科接受格菲妥单抗联合维泊妥珠单抗治疗的CAR-T细胞治疗失败的9例R/R DLBCL患者，对其临床资料进行回顾性分析。纳入标准：患者年龄达到18岁及以上，经组织学确诊为DLBCL，既往接受CAR-T细胞治疗失败且均未接受过维泊妥珠单抗、格菲妥单抗及其他CD20×CD3双抗治疗。排除标准则包括中枢神经系统淋巴瘤、HIV阳性的DLBCL、病历资料不完整或失访。所有病例数据均完成去标识化处理，本研究符合本院伦理审查豁免条件，豁免患者知情同意（批件号：IIT2026080015），研究过程严格道循《赫尔辛基宣言》伦理准则。

2. 治疗方案：给予奥妥珠单抗1 000 mg预处理，第1个周期第1天；格菲妥单抗2.5 mg，第8天；格菲妥单抗10 mg，第15天。第2个周期开始每个周期第1天给予格菲妥单抗剂量为30 mg。为降低细胞因子释放综合征（CRS）发生风险，患者在格菲妥单抗输注前1 h接受地塞米松20 mg、苯海拉明20 mg、复方对乙酰氨基酚250 mg的前驱用药。除疾病进展或出现不可管理的不良反应外，患者最多接受格菲妥单抗12个周期治疗。每个周期第1天给予维泊妥珠单抗1.8 mg/kg，最多6个周期。28 d为1个周期。

3. 疗效和不良反应评估：疗效评价采用2014版Lugano会议修订的淋巴瘤疗效评估标准，在治疗第2、4、6和12个周期后通过PET-CT进行评估疗效。总缓解率（ORR）为部分缓解（PR）率与完全缓解（CR）率的总和。主要不良事件包括CRS，其他不良事件分级参考美国卫生及公共服务不良事件评价标准（CTCAE）5.0版进行评估。

4. 随访：通过住院病历、门诊就诊记录和电话对患者进行定期随访，随访截止日期为2026年4月31日。无进展生存（PFS）期定义为自联合治疗开始至疾病进展、复发、任何原因导致的死亡或末次随访的时间；总生存（OS）期定义为自联合治疗开始至任何原因导致的死亡或末次随访的时间。

5. 统计学处理：计数资料采用频数和百分比（％）表示。计量资料采用*M*（范围）或*x*±*s*表示。所有的统计分析均使用SPSS 23.0版本软件完成。采用Kaplan-Meier生存曲线描述PFS和OS。*P*<0.05为差异具有统计学意义。

## 结果

1. 基线特征：如表1所示，9例患者中男4例、女5例，中位年龄69（49～75）岁。患者均为DLBCL，其中非生发中心起源（non-GCB）型6例。IPI评分4分4例、5分2例；Ann Arbor分期Ⅳ期6例。所有患者在CAR-T细胞治疗后进展时均伴有LDH升高，6例伴有结外病变，3例（例3、5、8）伴有大肿块（直径≥7.5 cm）。

**表1:** 9例复发/难治弥漫大B细胞淋巴瘤患者基线特征

例号	性别	年龄（岁）	ECOG评分（分）	IPI评分（分）	Ann Arbor分期	病理分型	结外受累部位	既往治疗线数	既往CAR-T细胞治疗最佳疗效	CAR-T细胞治疗失败类型	CAR-T细胞治疗缓解后至进展时间（月）
1	男	68	0	4	Ⅳ期	nGCB型	骨髓、浆膜腔	4	CR	缓解后复发	14
2	女	72	2	5	Ⅳ期	GCB型	骨髓、肾上腺	3	CR	缓解后复发	18
3	女	49	0	3	Ⅲ期	nGCB型	无	5	NR	原发难治	–
4	男	68	0	4	Ⅳ期	nGCB型	骨髓、肺	3	NR	原发难治	–
5	男	70	2	5	Ⅳ期	GCB型	骨髓、浆膜腔	3	CR	缓解后复发	16
6	女	71	2	4	Ⅲ期	nGCB型	无	4	CR	缓解后复发	15
7	女	67	0	3	Ⅲ期	nGCB型	无	5	CR	缓解后复发	13
8	女	75	1	4	Ⅳ期	GCB型	骨髓、胃肠道	3	CR	缓解后复发	17
9	男	69	0	3	Ⅳ期	nGCB型	骨髓	4	PR	缓解后复发	11

**注** ECOG：美国东部肿瘤协作组；IPI：国际预后指数；CAR-T细胞：嵌合抗原受体T细胞；nGCB：非生发中心起源；GCB：生发中心起源；CR：完全缓解；NR：未缓解；PR：部分缓解；–：患者为CAR-T细胞治疗原发难治

2. 疗效及预后：6个周期后通过PET-CT评估ORR为77.8％，其中CR率为55.6％，PR率为22.2％（图1）。2例患者因淋巴瘤进展而死亡，其中1例患者接受2个周期治疗后无效，疾病进展死亡；另1例患者接受8个周期治疗达到PR后出现疾病进展死亡。末次随访时5例患者为CR、2例患者为PR、2例患者死亡。中位随访12（5～18）个月时，中位PFS期及OS期均未达到。1年PFS率和OS率分别为71.1％（95％ *CI*：56.3％～86.1％）和76.2％（95％ *CI*：64.0％～94.2％）。

**图1:**
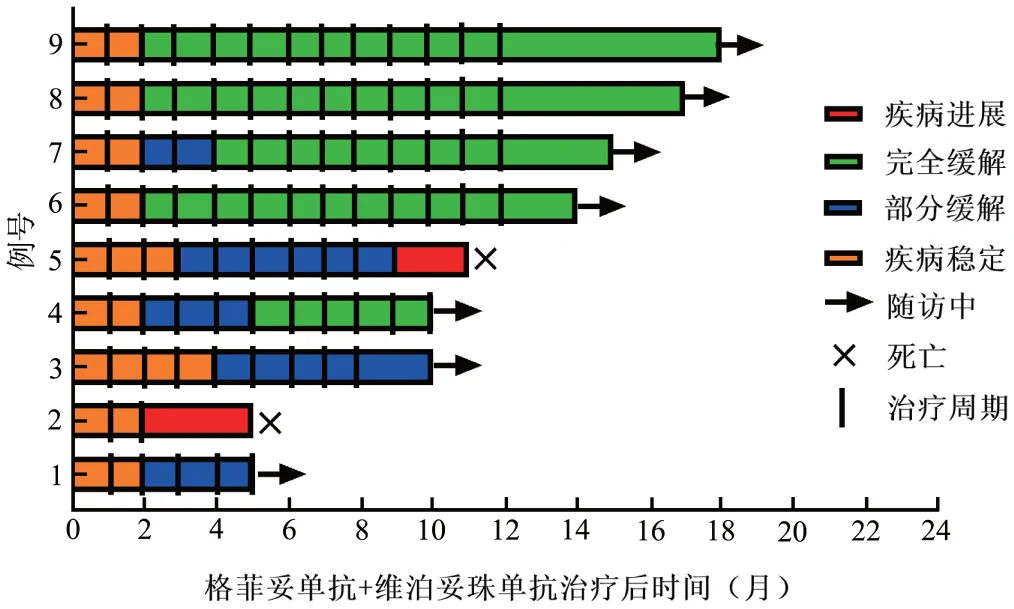
9例嵌合抗原受体T细胞治疗失败的复发/难治弥漫大B细胞淋巴瘤患者接受格菲妥单抗联合维泊妥珠单抗治疗疗效情况

3. 不良反应：格菲妥单抗联合维泊妥珠单抗治疗的总体耐受性良好，无患者因治疗相关不良事件而停止或减量治疗。治疗期间出现的所有不良事件见表2。9例患者均发生CRS，其中7例为1～2级、2例为3级。CRS中位发生时间为联合治疗后2（1～4）d，中位持续时间为5（3～11）d。3级CRS患者接受氧气支持、血管升压药、地塞米松、托珠单抗治疗后均好转。1例患者发生1级免疫效应细胞相关神经毒性综合征，经观察后好转。血液学不良反应较为常见，4例3～4级中性粒细胞减少，中位持续时间5（3～9）d，且均发生中性粒细胞缺乏伴发热；2例3～4级贫血，中位持续时间7（6～8）d；2例3～4级血小板减少，中位持续时间11（10～12）d。1例患者在首次使用格菲妥单抗后出现肿瘤溶解综合征的症状，表现为发热、低氧血症、呕吐、腹泻和腹痛，持续2 d，经积极补液、纠正电解质异常、抗高尿酸血症治疗和支持性治疗后好转。3例患者检测出至少1种明确病原体的感染，其中1例为新型冠状病毒感染，该患者接受奈玛特韦/利托那韦及丙种球蛋白治疗后好转；1例为流感嗜血杆菌感染，该患者接受头孢哌酮舒巴坦静脉输注抗感染治疗后好转；1例为屎肠球菌、烟曲霉混合性感染，该患者接受万古霉素联合伏立康唑治疗后好转。

**表2:** 格菲妥单抗联合维泊妥珠单抗治疗CAR-T细胞治疗失败的复发/难治DLBCL患者不良反应发生情况

不良反应	例（％）
CRS	
1～2级	7（77.8）
3～4级	2（22.2）
1～2级ICANS	1（11.1）
血液系统异常	
3～4级中性粒细胞减少	4（44.4）
3～4级贫血	2（22.2）
3～4级血小板减少	2（22.2）
肿瘤溶解综合征	1（11.1）
周围神经病变	2（22.2）
感染	3（33.3）
恶心	7（77.8）
呕吐	3（33.3）
腹泻	2（22.2）
便秘	5（55.5）
乏力	9（100）
头晕	1（11.1）
脱发	6（66.7）

**注** CAR-T细胞：嵌合抗原受体T细胞；DLBCL：弥漫大B细胞淋巴瘤；CRS：细胞因子释放综合征；ICANS：免疫效应细胞相关神经毒性综合征

## 讨论

本研究数据显示格菲妥单抗联合维泊妥珠单抗治疗9例CAR-T细胞治疗失败的R/R DLBCL患者，6个周期CR率为55.6％。一项纳入57例CAR-T细胞治疗后进展/复发的R/R DLBCL患者的研究中，采用维泊妥珠单抗联合BR方案CR率仅为14％[12]。既往研究证明双抗类药物对CAR-T细胞治疗失败的R/R DLBCL患者取得了一定疗效。一项单臂扩大队列研究表明，26例接受莫妥珠单抗单药治疗的既往接受过CAR-T细胞治疗的R/R DLBCL患者CR率为12％[13]。Bannerji等[14]报告30例接受奥尼妥单抗治疗的CAR-T细胞治疗失败的R/R DLBCL患者CR率为27％。Cartron等[15]报告格菲妥单抗治疗46例CAR-T细胞治疗失败的DLBCL患者CR率为45.7％。既往报道CAR-T细胞治疗失败的R/R DLBCL患者中位OS期短（分别为161 d和180 d）[6],[16]。

本研究结果显示格菲妥单抗联合维泊妥珠单抗治疗能够延长患者生存期，1年PFS率可达到71.1％。与单用双抗、单用维泊妥珠单抗方案、放疗或挽救化疗相比，联合方案作为一种固定疗程、现成可用、无化疗的方案，具有高缓解率、持久的缓解效果以及可控的安全性。同时，在本研究的有限样本中，未观察到既往CAR-T细胞疗效与本联合治疗反应之间的明确相关性，既往CAR-T细胞不同疗效状态的患者均可获得一定缓解，但仍需更大样本研究进一步验证。

治疗期间，患者总体耐受性良好。尽管所有患者均出现CRS，但经对症支持治疗后均好转，无治疗相关死亡事件。血液学不良反应较为常见。3例患者检测到了明确的病原体感染，经抗感染治疗后均好转。

总之，对于接受CAR-T细胞治疗失败的R/R DLBCL患者，格菲妥单抗联合维泊妥珠单抗的治疗方案显示了初步疗效及可控的安全性。本研究为回顾性、小样本量研究且随访时间较短，可能会出现选择偏倚等因素影响结论的准确性，患者的长期预后和生活质量仍在观察中，未来需要更大样本量的随机、前瞻性、对比性研究来验证。
